# Neck and waist circumference values according to sex, age, and body-mass index: Brazilian Longitudinal Study of Adult Health (ELSA-Brasil)

**DOI:** 10.1590/1414-431X20209815

**Published:** 2020-08-17

**Authors:** B.A.B. Moura, I.S. Santos, A.C. Goulart, M.I. Schmidt, P.A. Lotufo, I.M. Bensenor, C.P. Baena

**Affiliations:** 1 Escola de Medicina, Programa de Pós Graduação em Ciências da Saúde, Pontifícia Universidade Católica Paraná, Curitiba, PR, Brasil; 2 Centro de Pesquisa Clínica e Epidemiológica, Hospital Universitário, Universidade de São Paulo, São Paulo, SP, Brasil; 3 Programa de Pós-Graduação em Epidemiologia, Universidade Federal do Rio Grande do Sul, Porto Alegre, RS, Brasil

**Keywords:** Nomograms, Adiposity, Body-mass index, Waist, Neck

## Abstract

Body fat distribution predicts cardiovascular events better than body-mass index (BMI). Waist circumference (WC) and neck circumference (NC) are inexpensive anthropometric measurements. We aimed to present the conditional distribution of WC and NC values according to BMI, stratified by age and sex, from the Brazilian Longitudinal Study of Adult Health (ELSA-Brasil) baseline data. We analyzed 15,085 ELSA-Brasil participants with complete data. We used spline quantile regression models, stratified by sex and age, to estimate the NC and WC quantiles according to BMI. To test a putative association between age and median NC or WC values, we built sex-specific median regression models using both BMI and age as explanatory variables. We present estimated 25th, 50th, 75th, and 90th percentiles for NC and WC values, according to BMI, age, and sex. Predicted interquartile intervals for NC values varied from 1.6 to 3.8 cm and, for WC values, from 5.1 to 10.3 cm. Median NC was not associated with age in men (P=0.11) nor in women (P=0.79). However, median WC increased with advancing age in both sexes (P<0.001 for both). There was significant dispersion in WC and NC values for a given BMI and age strata for both men and women. WC, but not NC values, were associated with increasing age. The smaller influence of advancing age on the relationship between BMI and NC (compared to WC) values may be useful in longitudinal studies.

## Introduction

The prevalence of obesity is increasing worldwide and a growing body of evidence shows that body fat distribution might add important information for predicting cardiovascular events above and beyond body-mass index (BMI) itself (1).

A seminal work published in the 1950s already reported that differences in the localization of adiposity between men and women are linked to their different cardiovascular profile (2). The study of fat tissue distribution and cardiovascular risk has gained more attention recently, and it has been hypothesized that specific fat depots could increase vascular damage (3,4), through mediators that can influence glucose homeostasis and lipid metabolism, inflammation, and coagulation (4). Certain locations of fat accumulation have been linked to diverse cardiometabolic profiles (5), suggesting that regional fat distribution could play an important role in the development of cardiovascular diseases in both non-obese and obese people (5,6). Recently, the Consensus Statement of the International Atherosclerosis Society (7) argued for the inclusion of waist circumference (WC) as a vital sign given that the prevalence of abdominal obesity is increasing and dysfunctional adipose tissue could be estimated more accurately by WC than BMI as shown in recent studies. Additionally, the consensus point out for the gap in knowledge with a recommendation for description of WC values for a given BMI category across different ages, by sex, as the limitations of BMI have been increasingly demonstrated in different populations especially in demonstrating changes in adiposity during aging (8,9). Moreover, it is difficult to measure body fat mass directly, while WC and neck circumference (NC) are inexpensive and easily obtainable anthropometric measurements (7,10).

Another study looked at one Brazilian population of the Baependi Heart Study in a cross-sectional analysis and showed that WC in men discriminated the hypertensives better than visceral and body adiposity indexes (11). Body fat distribution patterns vary, and the positive correlations between BMI and both NC and WC do not follow perfect linear associations, and despite being associated with higher cardiovascular risk in different populations (12–15), factors as sex, age, physical activity, smoking habits, number of pregnancies, and genetic predisposition have also been linked to body fat distribution (16–18)

Hingorjo et al. (19) studied 150 young university students in Pakistan and found that approximately 30% of NC variance in males and 50% of NC variance in females was not explained by BMI values. In addition, these discrepancies may vary in different populations, and in different time periods. Stern et al. (20) showed that the predicted WC according to BMI in Chinese men and women increased from 1993 to 2009. In the same country and time interval, Du et al. (21) reported that the prevalence of central obesity in adults with normal (<25 kg/m^2^) BMI increased from 11.9 to 21.1% during 16 years.

This underlines the importance of studying the WC and NC values according to BMI in large epidemiologic studies, conducted within different populations. To date, there is no such study conducted in large samples. Therefore, our aim was to present the conditional distribution of WC and NC values according to BMI, stratified by age and sex, among mid-adult and elderly men and women participants at the baseline assessment of the Brazilian Longitudinal Study of Adult Health (ELSA-Brasil) study, a large multicenter cohort study in Brazil.

## Material and Methods

### Study design

ELSA-Brasil is a multicenter prospective cohort study (22,23) that enrolled 15,105 civil servants aged 35 to 74 years from 6 Brazilian cities (Belo Horizonte, Porto Alegre, Rio de Janeiro, Salvador, São Paulo, and Vitória) (24). In this cross-sectional analysis, we used baseline data (2008–2010). Approvals were obtained from the institutional review boards of all the centers, and all the subjects signed an informed consent form.

### Study sample

From 15,105 ELSA-Brasil participants at baseline, we excluded 20 (0.1%) that did not have complete BMI, WC, or NC data. Our sample comprised 6,879 (45.6%) men and 8,206 (54.4%) women with complete data.

### Study variables

Height and weight were measured using a standardized scale and a fixed stadiometer, and BMI was calculated by dividing body weight by the squared height in meters (kg/m^2^). WC was measured using an inelastic tape of 150 cm (Mabis-Gulick, USA) at the midpoint between the lowest rib margin and the iliac crest (10). NC was measured with an inelastic tape (mm) right under the thyroid cartilage and perpendicular to the long axis of the neck, with the participant in a sitting position. All measurements were performed by trained nurses. The intra-class correlation coefficient for repeated measurements was 0.98 (95%CI: 0.85–1.0) (25).

Age is presented as a continuous variable and also stratified as 35–44 years, 45–54 years, 55–64 years, and 65–74 years. Race was self-reported as White, Brown, Black, Asian, and Native. Educational level was stratified as up to incomplete high school, high-school, and college or above. Smoking status was self-reported as never, former, and current smoker. Monthly family income at baseline was converted from Brazilian reals (BRL$) to US dollars (USD$) at a rate USD$1 = BRL$2 and stratified as <USD$1,245, USD$1245-3,319, and ≥USD$3,320. Excessive alcohol drinking was defined as >210 g/week for men and >140 g/week for women. Blood pressure was obtained in the sitting position after a minimum rest period of 5 min. Three consecutive readings were obtained for each participant, after one-minute interval between each one. The mean of the two last measurements was defined as the casual blood pressure.

Laboratory measurements were obtained after an overnight fast. Fasting glucose was determined enzymatically by the hexokinase method. Total cholesterol, high-density cholesterol (HDL-cholesterol), low-density cholesterol (LDL-cholesterol), and triglycerides were determined by the enzymatic colorimetric method (22,24,25). Hypertension was defined as the use of medications to treat hypertension, systolic blood pressure ≥140 mmHg, or diastolic blood pressure ≥90 mmHg at baseline. Diabetes was defined by a medical history of diabetes, use of medications to treat diabetes, a fasting glucose ≥126 mg/dL, glycated hemoglobin (HbA1C) levels ≥6.5%, or a 2-h oral glucose tolerance test ≥200 mg/dL. Dyslipidemia was defined as use of lipid-lowering treatment or a LDL cholesterol level ≥130 mg/dL.

### Statistical analysis

Categorical variables are reported as absolute counts and proportions. Continuous variables are reported as means±SD or median (interquartile range). We used spline quantile regression models, stratified by sex and age, to estimate the conditional distribution of NC and WC according to BMI. These models were used to estimate the 25th, 50th, 75th, and 90th percentiles for NC and WC values, in the BMI range between 20 and 40 kg/m^2^. To test a putative association between age and median NC or WC values in men and women, we built sex-specific median regression models using both BMI and age as explanatory variables. Analyses were performed using the R software. Significance level was set at 0.05.

## Results

**Figure 1:**
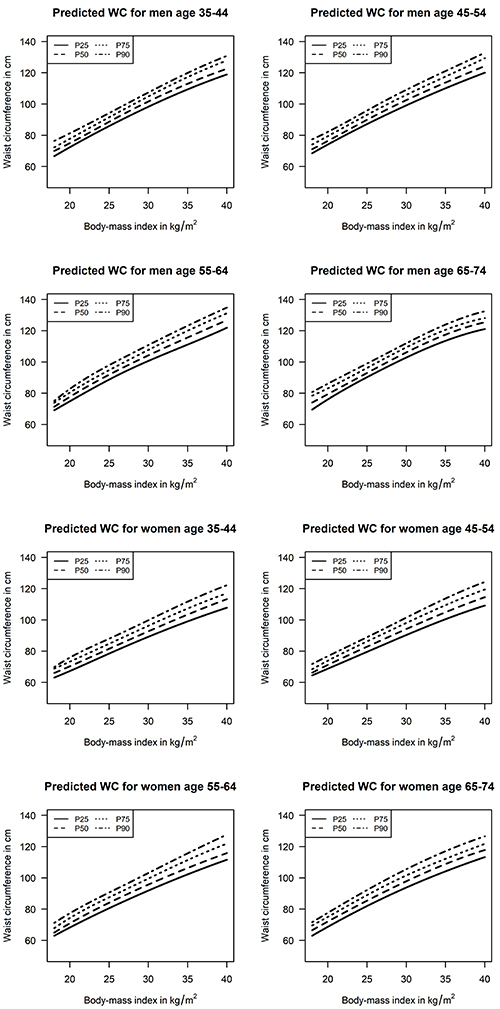
Predicted 25th, 50th, 75th, and 90th percentiles for waist circumference (WC) values according to age, body mass index, and sex.

**Figure 2:**
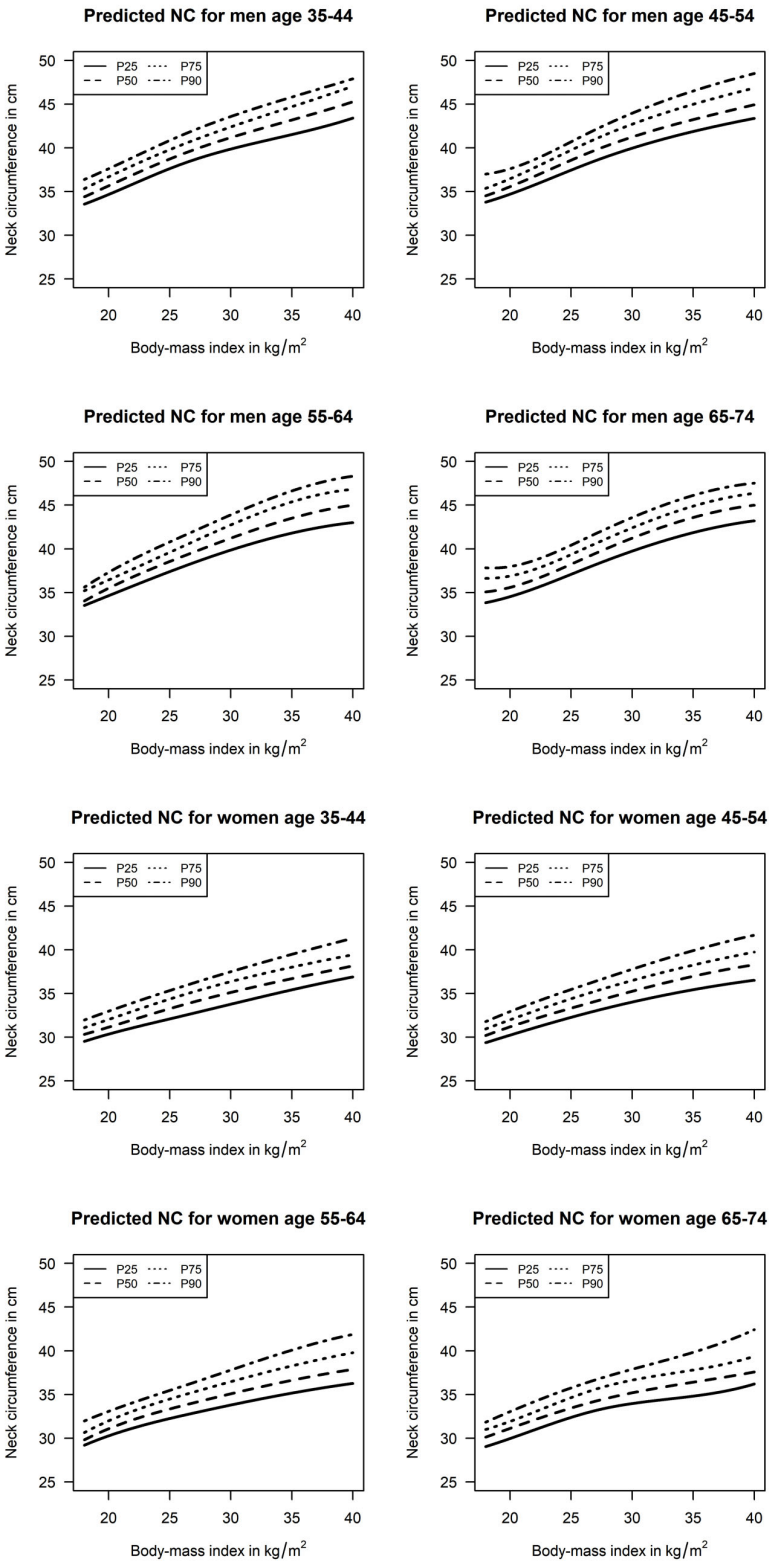
Predicted 25th, 50th, 75th, and 90th percentiles for neck circumference (NC) values according to age, body mass index, and sex.

Table 1 details the characteristics of the sample according to sex. The mean age was 52.1 years. Most of the participants self-reported being of White race (52.2%), having a college education (52.6%), and never having smoked (56.9%). Table 2 shows the estimated 25th, 50th, 75th, and 90th percentiles for NC values (in cm), according to BMI, age strata, and sex. Predicted interquartile intervals (75th-25th percentile) for NC values varied from 1.6 to 3.8 cm (5.0 to 9.2% of predicted median values). Similarly, Table 3 shows the predicted conditional distribution for WC values (in cm), also according to BMI, age strata, and sex. Predicted interquartile intervals for WC values varied from 5.1 to 10.3 cm (5.6 to 9.0%) of predicted median values). Graphical presentations of the results are available in Figures 1 and 2.

**Table 1:** Characteristics of the study sample.

	Men(N=6879)	Women(N=8206)	All(N=15085)
Age (years; mean±SD)	52.2±9.3	52.0±8.9	52.1±9.1
35–44 years (N, %)	1559 (22.7%)	1779 (21.7%)	3338 (22.1%)
45–54 years (N, %)	2681 (39.0%)	3250 (39.6%)	5931 (39.3%)
55–64 years (N, %)	1852 (26.9%)	2374 (28.9%)	4226 (28.0%)
65–74 years (N, %)	787 (11.4%)	803 (9.8%)	1590 (10.5%)
Race			
White (N, %)	3596 (53.0%)	4187 (51.6%)	7783 (52.2%)
Brown (N, %)	2026 (29.9%)	2171 (26.7%)	4197 (28.2%)
Black (N, %)	939 (13.8%)	1454 (17.9%)	2393 (16.1%)
Other (N, %)	220 (3.2%)	310 (3.8%)	530 (3.6%)
Educational level			
Incomplete high school (N, %)	1138 (16.5%)	783 (9.5%)	1921 (12.7%)
High school (N, %)	2268 (33.0%)	2959 (36.1%)	5227 (34.7%)
College or above (N, %)	3473 (50.5%)	4464 (54.4%)	7937 (52.6%)
Monthly family income			
<USD1245 (N, %)	1808 (26.4%)	2182 (26.7%)	3990 (26.6%)
USD1245-3319 (N, %)	2848 (41.6%)	3770 (46.2%)	6618 (44.1%)
≥USD3320 (N, %)	2195 (32.0%)	2216 (27.1%)	4411 (29.4%)
Hypertension (N, %)	2756 (40.1%)	2637 (32.2%)	5393 (35.8%)
Diabetes (N, %)	1597 (23.2%)	1363 (16.6%)	2960 (19.6%)
Dyslipidemia (N, %)	4063 (59.2%)	4702 (57.3%)	8765 (58.2%)
Smoking			
Never (N, %)	3460 (50.3%)	5117 (62.4%)	8577 (56.9%)
Past (N, %)	2434 (35.4%)	2096 (25.5%)	4530 (30.0%)
Current (N, %)	984 (14.3%)	993 (12.1%)	1977 (13.1%)
Excessive drinking (N, %)	835 (12.2%)	287 (3.5%)	1122 (7.5%)
Systolic blood pressure (mmHg; mean±SD)	125.6±16.8	117.7±16.9	121.3±17.3
Diastolic blood pressure (mmHg; mean±SD)	78.9±10.8	74.0±10.2	76.2±10.8
Body-mass index (kg/m^2^; mean±SD)	27.0±4.3	27.1±5.1	27.0±4.7
Neck circumference (cm; mean±SD)	39.5±2.9	34.0±2.6	36.5±3.9
Waist circumference (cm; mean±SD)	95.3±11.7	87.8±12.6	91.2±12.8
Fasting plasma glucose (mg/dL; mean±SD)	116.6±34.1	108.0±27.2	111.9±30.8
Total cholesterol (mg/dL; mean±SD)	212.6±44.2	216.5±41.2	214.7±42.7
LDL-cholesterol (mg/dL; mean±SD)	130.8±35.9	131.2±34.5	131.0±35.1
HDL-cholesterol (mg/dL; mean±SD)	50.8±12.2	61.6±14.6	56.7±14.6
Triglycerides (mg/dL; median [P25-P75])	132.0 [93.0-192.0]	103.0 [75.0-144.0]	115.0 [82.0-166.0]

LDL: low-density cholesterol; HDL: high-density cholesterol.

**Table 2:** Neck circumference predicted quantiles for sex, age, and body mass index (BMI).

Age	BMI	Men				Women			
		P25	P50	P75	P90	P25	P50	P75	P90
35–44 years	20.0	34.7	35.7	36.7	37.6	30.4	31.2	32.0	33.0
	22.5	36.2	37.2	38.3	39.3	31.3	32.2	33.2	34.2
	25.0	37.6	38.7	39.8	40.9	32.1	33.3	34.4	35.3
	27.5	38.9	40.0	41.2	42.3	32.9	34.2	35.4	36.4
	30.0	39.9	41.2	42.4	43.6	33.8	35.1	36.4	37.5
	32.5	40.7	42.2	43.6	44.7	34.6	35.9	37.2	38.5
	35.0	41.5	43.2	44.7	45.8	35.4	36.7	38.0	39.5
	37.5	42.4	44.2	45.9	46.9	36.2	37.4	38.8	40.4
	40.0	43.4	45.3	47.1	47.9	36.9	38.2	39.4	41.3
45–54 years	20.0	34.7	35.6	36.5	37.6	30.2	31.2	32.0	32.9
	22.5	36.0	37.1	38.1	39.0	31.3	32.3	33.2	34.3
	25.0	37.5	38.6	39.7	40.7	32.3	33.3	34.4	35.5
	27.5	38.8	40.0	41.3	42.4	33.2	34.3	35.5	36.6
	30.0	40.0	41.2	42.7	44.0	34.0	35.3	36.5	37.8
	32.5	41.0	42.3	43.9	45.3	34.8	36.2	37.4	38.9
	35.0	41.9	43.2	45.0	46.5	35.4	37.0	38.3	39.9
	37.5	42.7	44.1	46.0	47.6	36.0	37.7	39.0	40.9
	40.0	43.4	44.9	46.9	48.5	36.5	38.3	39.7	41.7
55–64 years	20.0	34.6	35.5	36.4	37.3	30.3	31.1	32.0	33.1
	22.5	36.0	37.1	38.0	39.2	31.4	32.3	33.4	34.3
	25.0	37.4	38.6	39.6	40.8	32.3	33.3	34.5	35.5
	27.5	38.7	39.9	41.2	42.4	33.0	34.2	35.5	36.6
	30.0	39.9	41.2	42.7	43.9	33.8	35.1	36.5	37.8
	32.5	40.9	42.4	44.2	45.3	34.5	35.9	37.4	39.0
	35.0	41.8	43.5	45.4	46.6	35.2	36.6	38.3	40.1
	37.5	42.5	44.4	46.3	47.6	35.7	37.3	39.1	41.1
	40.0	43.0	45.0	46.8	48.3	36.3	37.9	39.8	41.9
65–74 years	20.0	34.5	35.6	36.9	38.0	30.0	31.1	31.9	33.0
	22.5	35.7	36.8	37.9	38.9	31.2	32.3	33.3	34.5
	25.0	37.1	38.2	39.4	40.4	32.4	33.5	34.6	35.8
	27.5	38.5	39.8	40.9	42.1	33.3	34.4	35.8	36.9
	30.0	39.7	41.2	42.4	43.6	34.0	35.2	36.6	37.9
	32.5	40.9	42.5	43.7	45.0	34.4	35.8	37.3	38.8
	35.0	41.9	43.6	44.9	46.1	34.8	36.4	37.8	39.8
	37.5	42.6	44.4	45.8	47.0	35.4	37.0	38.5	41.0
	40.0	43.2	45.0	46.4	47.5	36.2	37.6	39.4	42.4

**Table 3:** Waist circumference predicted quantiles for sex, age, and body mass index (BMI).

Age	BMI	Men				Women			
		P25	P50	P75	P90	P25	P50	P75	P90
35–44 years	20.0	72.4	75.0	77.8	81.3	67.3	70.4	73.3	75.9
	22.5	79.3	81.8	84.7	87.7	72.9	75.9	79.0	82.3
	25.0	85.9	88.7	91.5	94.3	78.5	81.5	84.8	88.0
	27.5	92.3	95.4	98.3	100.9	84.0	87.2	90.5	93.8
	30.0	98.3	101.6	104.9	107.4	89.2	92.7	96.3	99.8
	32.5	104.0	107.5	111.2	113.8	94.3	98.2	101.9	105.9
	35.0	109.4	113.0	117.3	119.9	99.1	103.4	107.3	111.7
	37.5	114.4	118.0	122.8	125.6	103.6	108.5	112.4	117.2
	40.0	118.9	122.6	127.9	130.8	107.8	113.3	117.1	122.2
45–54 years	20.0	74.1	76.4	79.5	82.2	68.8	71.2	73.9	76.8
	22.5	80.8	83.2	86.3	88.9	74.2	77.1	80.3	82.9
	25.0	87.2	90.0	93.1	95.9	79.6	82.9	86.3	89.0
	27.5	93.3	96.5	99.7	102.7	85.0	88.6	92.2	95.2
	30.0	99.2	102.5	105.9	109.1	90.4	94.2	98.1	101.5
	32.5	104.8	108.3	111.8	115.2	95.6	99.7	103.9	107.8
	35.0	110.1	113.8	117.6	121.2	100.5	105.0	109.5	113.8
	37.5	115.2	119.1	123.4	127.0	105.1	109.9	114.7	119.3
	40.0	120.1	124.3	129.3	132.8	109.2	114.6	119.5	124.3
55–64 years	20.0	74.9	77.7	80.5	82.8	68.3	70.9	74.3	77.4
	22.5	82.0	85.1	88.0	91.0	74.6	77.8	81.3	84.3
	25.0	88.8	91.9	94.8	98.0	80.6	84.1	87.6	90.6
	27.5	95.0	98.2	101.3	104.5	86.4	90.0	93.5	96.8
	30.0	100.7	104.3	107.7	110.9	91.9	95.7	99.6	103.1
	32.5	106.0	110.0	114.0	117.1	97.2	101.2	105.6	109.5
	35.0	111.2	115.7	120.0	123.2	102.2	106.3	111.4	115.7
	37.5	116.4	121.2	125.7	129.1	107.1	111.2	116.9	121.8
	40.0	121.9	126.8	131.2	134.8	111.6	115.9	121.9	127.7
65–74 years	20.0	76.1	79.4	83.1	86.1	68.7	72.1	75.1	77.6
	22.5	83.5	86.2	89.5	92.6	75.5	78.9	82.1	85.1
	25.0	90.3	93.0	96.2	99.0	82.0	85.4	89.0	92.2
	27.5	96.8	99.7	102.9	105.5	88.1	91.8	95.6	99.1
	30.0	102.9	106.1	109.4	112.0	93.8	97.8	101.6	105.5
	32.5	108.5	112.2	115.4	118.3	99.2	103.6	107.1	111.6
	35.0	113.6	117.5	120.6	124.0	104.2	108.9	112.2	117.1
	37.5	117.8	122.0	124.9	128.8	108.9	113.7	117.1	122.1
	40.0	121.1	125.4	128.1	132.5	113.3	117.8	121.8	126.6

**Figure 3:**
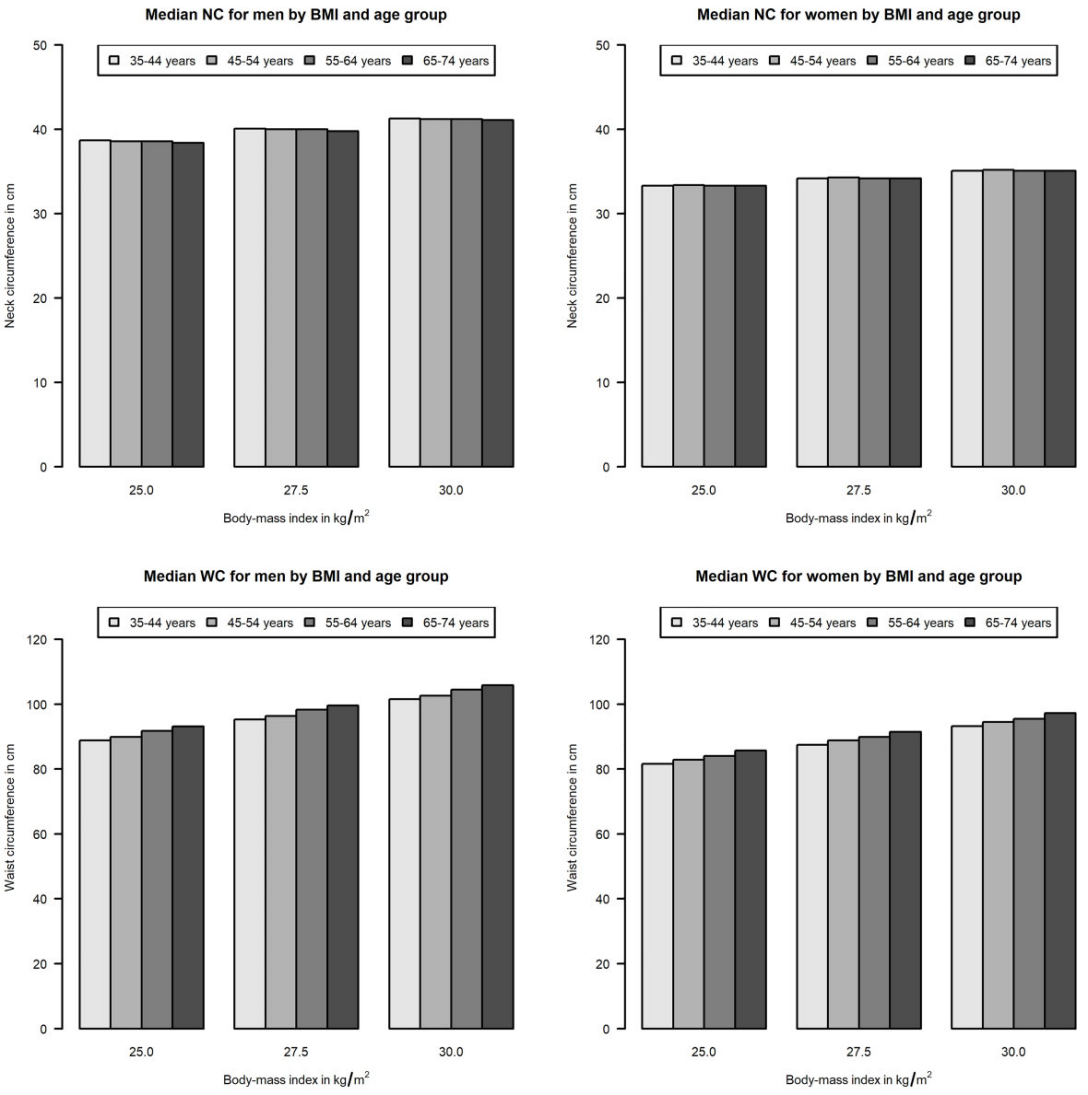
Median waist (WC) and neck circumference (NC) values according to age strata, for men and women with body mass index of 25.0, 27.5, and 30.0 kg/m^2^. Data are reported as means.

We built sex-specific median regression models using both BMI and age as explanatory variables to test if median NC or WC were associated with age in men and women. We found median NC was not associated with age in men (P=0.11) nor women (P=0.79). However, median WC increased with advancing age in both sexes (P<0.001 for both). Figure 3 shows predicted median WC and NC values for men and women with BMIs of 25.0, 27.5, and 30.0 kg/m^2^.

## Discussion

We presented the conditional distribution of WC and NC values, according to age, sex, and BMI values in a large sample of Brazilian adults. There was a significant variance in WC and NC values for a given BMI and age strata for both men and women. In addition, we found that WC, but not NC values, were associated with increasing age. Some mechanisms have been proposed to explain different fat tissue distribution within individuals with the same BMI, such as dysfunctional adipose tissue, sedentary lifestyle, or both (26). As mentioned above, local body fat mass and its clinical markers, as NC and WC, are associated with multiple phenotypes of higher cardiovascular risk. The association between these phenotypes and NC or WC cannot be explained exclusively by higher BMIs. Evidence from the Framingham Study shows that body fat distribution and fat depots could be better predictors of cardiovascular diseases (CVD) than BMI (27). Population data from the European Prospective Investigation into Cancer and Nutrition of the Norfolk cohort showed that WC and waist-to-hip ratio were more consistent predictors of coronary heart disease than BMI (1). The results of the Framingham Heart Study showed that NC was associated with CVD risk factors after adjustment for BMI (6). In addition, for specific scenarios, the association between these anthropometric measurements and cardiovascular risk may be heterogeneous, or even additive. In a comparison of the clinical usefulness of NC and WC in individuals with severe obesity (mean BMI 36.9; mean age 49 years), NC values had stronger associations with type 2 diabetes, insulin resistance, metabolic syndrome, and hypertension compared to WC values (28). In the ELSA-Brasil, NC was significantly associated with cardio-metabolic risk factors as insulin resistance, hypertriglyceridemia, and higher blood pressure after adjustment for WC and BMI (14,15).

The study of body fat distribution patterns in subjects with similar BMI may be important for both identifying individuals at a higher cardiovascular risk (compared to peers with the same BMI) and understanding the factors that lead to unfavorable fat distribution profiles. There is evidence that WC values are increasing more than expected for the increase in BMI values in recent decades in different populations. Stern et al. (20) analyzed data from 6,159 Chinese men and women aged 20 to 59 years in 1993 and 6,644 Chinese men and women with the same age range in 2009. They found that for every age strata and in both sexes, predicted WC for individuals with a BMI of 25 or 28 kg/m^2^ were higher in 2009 than in 1993. Janssen et al. (29) compared data from 15,688 subjects aged 7 to 69 years in 1981 to 4,987 individuals, also aged 7 to 69 years, who were evaluated in 2007–2009 in Canada. They found that for individuals with a BMI of 25 kg/m^2^, the predicted WC values in 2007–2009 were 1 to 5 cm higher than in 1981. In addition, each 1 kg/m^2^ increase in BMI value was associated with higher WC increases in 2007–2009 compared to 1981. Walls et al (30) compared NHANES data from 1988–1994 (15,349 participants) and 2005–2006 (4,176 participants) and found that WC values in American adults younger than 50 years of age (but not in older individuals) increased 0.9 cm more than expected for the rise in BMI values during this period. Another study with aggregated data from three cross-sectional surveys taken in 1989, 1999–2000, and 2011–2012 (n=8313, 5903, and 3904, respectively) looked at WC change in Australians and found an independent increase of WC, showing that the proportion of obese people detected by WC increased 10% for women and 6% for men (9).

On the other hand, Elobeid et al. (31) analyzed a different time-frame in the United States (1954–2004), and did not find a slope for the relationship between WC and BMI over time significantly different from zero. The relationship between NC and BMI is less studied and, to our knowledge, there are no large epidemiological studies describing the conditional distribution of NC values according to BMI and age strata. Our results highlight the importance of such descriptions, as we found that the relationship between BMI and NC values was influenced less by age strata than the relationship between BMI and WC values. Future longitudinal analysis of ELSA-Brasil data will provide important information about the clinical relevance of this finding.

A study by Stern et al. (20) shows predicted WC values for Chinese adults with a BMI of 25 kg/m^2^ and for those with a BMI of 28 kg/m^2^. We compared their 2009 data (which matches the inclusion period for ELSA-Brasil) to our predicted median WC values for men and women with the same BMI values. We found slightly higher predicted WC values for men and lower predicted WC values for women in ELSA-Brasil compared to the Chinese population. In all cases, estimates did not differ by more than 3 cm. Some differences between these two studies may be partially accountable for this finding. First, Stern et al. (20) used linear regression (which is a least square model for mean values) and in our study we used quantile regression (which is a linear mathematical optimization technique for estimating quantile values, including the median). Although we aimed to compare similar age strata, it is possible that heterogeneity in age stratification cutoffs may also have yielded different estimates, as both studies point to higher WC values according to BMI with increasing age.

Our study had some limitations. As it is a descriptive study with cross-sectional design, causal inferences were not focused. Although inexpensive, both WC and NC may be prone to measurement errors, and NC values may also be influenced by neck muscular volume. Therefore, measurements in other samples should be studied before using these values as a screening tool. As strengths, our study described the distribution of two anthropometric measurements in a very large multicenter epidemiologic study in Brazil. The conditional distribution of these values, according to BMI, may be used as markers of body fat distribution in future prospective ELSA-Brasil analyses. To our knowledge, analyses of large samples focusing on NC values distributions, conditioned to BMI and age, were not previously published. Although it must be confirmed by prospective data, the smaller influence of advancing age on the association between BMI and NC (compared to WC) values may be useful to help understand distribution of body fat in longitudinal studies. We believe our study contributes to fill the gap of evidence mentioned in the recent statement of the International Atherosclerosis Society (7), in terms of providing a description of two adiposity measures by different BMI, age, and sex. Moreover, our study adds to the previous body of evidence on the change of waist (9,17) and neck circumferences according to BMI, age, and sex as an easy and reproducible tool to identify adverse fat depots phenotypes.

In this study, we estimated sex- and age-specific quantile values for NC and WC according to BMI. There was significant dispersion in WC and NC values for a given BMI and age strata for both men and women. WC, but not NC values, were associated with increasing age.
